# Efficient IPv6 address scanning based on hostname correlation in IPv6-only network

**DOI:** 10.1038/s41598-026-39577-2

**Published:** 2026-02-13

**Authors:** Ce Sun, Liancheng Zhang, Ruijuan Wang, Yakai Fang, Hongtao Zhang, Haojie Zhu, Luyang Li

**Affiliations:** 1 School of Cyber Science and Engineering, Zhengzhou University, Zhengzhou, 450001 China; 2 Information Engineering University, Zhengzhou, 450001 China; 3 Key Laboratory of Cyberspace Security, Ministry of Education, Zhengzhou, 450001 China

**Keywords:** IPv6, Address scanning, Hostname correlation, On-link, IPv6-only, Computational biology and bioinformatics, Engineering, Mathematics and computing

## Abstract

Existing on-link IPv6 address scanning technologies based on IPv6-only information, such as multicast Ping6 scanning, invalid extension header scanning, multicast listener discovery (MLD) scanning, and stateless address auto-configuration scanning, rely on protocol features such as ICMPv6, MLD, and other protocol features to induce responses. However, these scanning packets are easily intercepted by the default security mechanisms of modern OSs (Operating Systems), leading to issues such as low OS coverage and incomplete IPv6 address scan results of alive nodes. On-link IPv6 address scanning technologies based on dual-stack correlation information (e.g., FScan6, LLMNR6, and LinkScan6) utilize local domain names, hostnames, and other dual-stack correlation information to enhance IPv6 address discovery capabilities. However, these technologies overly rely on IPv4 networks, resulting in low IPv6 address scanning efficiency and inability to operate in IPv6-only networks. To this end, we propose HFinder6, an efficient IPv6 address scanning technology for IPv6-only network based on hostname correlation. HFinder6 combines the passive capture of DHCPv6 Solicit messages from active IPv6 nodes with an active triggering mechanism. By constructing Router Advertisement messages of NDP protocol, it forces nodes to proactively send Solicit messages, enabling rapid hostname acquisition. Subsequently, the IPv6 address information is queried in parallel using the mDNS protocol and LLMNR protocol, thereby achieving efficient scanning of IPv6 addresses for alive nodes in IPv6-only network. A typical IPv6-only and IPv4/IPv6 dual-stack network environment was established, comprising 20 versions of OSs such as Windows and Linux. HFinder6 was tested in this environment and compared with 4 IPv6-only network IPv6 address scanning scripts from the Nmap tool and 3 dual-stack network IPv6 address scanning tools (e.g., LinkScan6, LLMNR6, and FScan6). Experimental results show that HFinder6 can discover 43 IPv6 addresses across 18 OS versions in an average of just 10.29 seconds within IPv6-only network. In terms of OS coverage and IPv6 address scanning completeness, HFinder6 performs on par with FScan6 and outperforms 6 other scripts and tools, successfully identifies at more 14 additional OS versions and scans 35 more IPv6 addresses, thereby enhancing the completeness of IPv6 address scanning by up to 5.37 times. Moreover, in terms of IPv6 address scanning efficiency, HFinder6 can identify 3.67 more IPv6 addresses per second, outperforming these 7 scripts and tools.

## Introduction

In response to the challenges of on-link IPv6 address scanning technologies, researchers have proposed two solution approaches. Early on-link IPv6 address scanning technologies^1–4^ based on IPv6-only information induced node responses by sending ICMPv6 Echo Request messages and ICMPv6 Echo Request messages with invalid extension headers, etc., faced two major challenges: Modern OS security mechanisms intercept abnormal probe messages, rendering address scanning ineffective and reducing the OS coverage of these technologies. Additionally, the multiple addresses of nodes, including permanent GUA address, temporary GUA address, and LLA address^5^, lead to incomplete IPv6 address scanning results.

To overcome these limitations, subsequent research proposed on-link IPv6 address scanning technologies based on dual-stack correlation information (e.g., LinkScan6^6^, LLMNR6^7^, and FScan6^8^), which first obtain host identifiers such as MAC addresses in IPv4 network and then use these information to obtain IPv6 address configurations. While these technologies mitigate low OS coverage and incomplete IPv6 address scanning results, they still exhibit notable limitations: The use of multiple probes reduces overall scanning efficiency, and their reliance on IPv4 information will become increasingly obsolete as networks continue transitioning to IPv6-only architectures. These limitations underscore the urgent need to develop new on-link IPv6 address scanning technologies based on IPv6-only information.

Existing on-link IPv6 address scanning technologies based on IPv6-only information suffer from insufficient OS coverage and incomplete IPv6 address acquisition. Meanwhile, on-link IPv6 address scanning technologies based on dual-stack correlation information face challenges regarding low efficiency and incompatibility with IPv6-only networks. To address these limitations, this paper proposes a hostname acquisition mechanism primarily based on DHCPv6, combining the broad applicability and “plug-and-play” characteristics of the DHCPv6 protocol with the NDP, mDNS, and LLMNR protocols to achieve a combination of active discovery and passive monitoring, supporting multiple OS nodes. Based on this mechanism, we present HFinder6, an efficient on-link IPv6 address scanning technology based on hostname correlation in IPv6-only network.

**Fig. 1:**
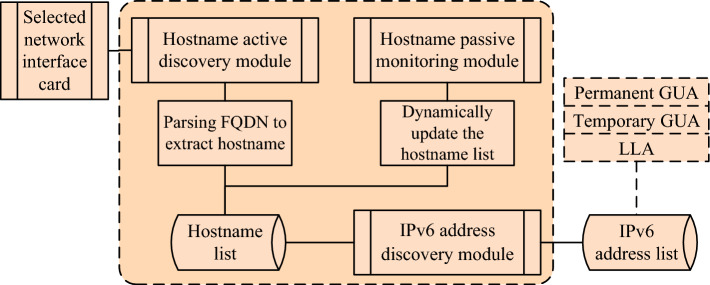
HFinder6 workflow diagram.

HFinder6 uses the hostname active discovery module to obtain hostname information of alive on-link hosts and add them to the hostname list. The hostname passive monitoring module continuously listens on the link and dynamically updates the hostname list. The IPv6 address discovery module uses the hostname list to construct probe messages to obtain the IPv6 address configuration of hosts corresponding to hostnames on-link, as shown in Fig. 1.

The main contributions of this paper are as follows:

- This study first actively discovers hostnames of alive on-link nodes through DHCPv6 Solicit messages, then leverages DHCPv6 protocol’s broad applicability and plug-and-play nature to achieve efficient IPv6 address scanning in IPv6-only network by parallel querying using both mDNS and LLMNR Query messages.
- This study innovatively proposes a hostname acquisition mechanism based on RA messages of NDP protocol to actively trigger DHCPv6 Solicit messages, solving the problem that the DHCPv6 protocol can only passively listen for hostnames and has a limited time window. This mechanism combines the NDP protocol with the DHCPv6 protocol for the first time, providing a new way to obtain hostnames for IPv6 address scanning.
- This research presents an innovative and efficient on-link IPv6 address scanning technology, employing a two-stage scanning framework based on hostname correlation to address the limitations of traditional on-link IPv6 address scanning technologies based on dual-stack correlation information. Compared to existing three-stage technologies, the proposed technology streamlines the scanning process, improving scanning efficiency by at least 60%. Theoretical analysis and experimental results confirm that this proposed technology operates independently of IPv4, providing an effective solution for IPv6 address scanning in IPv6-only networks.
- Designed and implemented an efficient on-link IPv6 address scanning tool, HFinder6, tailored for IPv6-only networks, integrating active discovery with passive monitoring. In a network environment comprising 20 different OSs, it was compared with 4 IPv6-only network IPv6 address scanning scripts from the Nmap tool and 3 dual-stack network IPv6 address scanning tools (e.g., LinkScan6, LLMNR6, and FScan6). In terms of OS coverage and IPv6 address scanning completeness, it outperforms most existing technologies and performs on par with the currently most advanced FScan6. Crucially, unlike FScan6, HFinder6 operates effectively in IPv6-only networks and achieves superior scanning efficiency.

“Introduction” provides an overview of on-link IPv6 address scanning technologies. “Analysis of on-link IPv6 address scanning technologies” introduces the related work and current research status of on-link IPv6 address scanning technologies. “Basics of IPv6 address scanning mechanism based on hostname correlation” introduces the basics of obtaining IPv6 addresses using hostname correlation. “Efficient IPv6 address scanning technology based on hostname correlation in IPv6-only network” introduces the efficient IPv6 address scanning technology based on hostname correlation in IPv6-only network proposed in this paper. “Comparative analysis and discussion of experimental results” verifies the comprehensiveness, speed, and effectiveness of the proposed technology through comparative experiments. “Limitations and ethical considerations” discusses technical constraints and operational safety. Finally, “Conclusion” summarizes the entire paper. For clarity, the abbreviations used throughout this paper are listed in Table 1.

**Table 1:** List of abbreviations and full terms.

Abbreviations	Full terms	Abbreviations	Full terms
IPv6/IPv4	Internet Protocol version 6/Internet Protocol version 4	RFC	Request for Comments
IEH	Invalid Extension Header	FQDN	Fully Qualified Domain Name
ICMPv6	Internet Control Message Protocol for IPv6	DHCP	Dynamic Host Configuration Protocol
OS	Operating System	SLD	Second-Level Domain
GUA	Global Unicast Address	TLD	Top-Level Domain
LLA	Link-Local Address	IP	Internet Protocol
MAC	Media Access Control	UDP	User Datagram Protocol
DHCPv6	Dynamic Host Configuration Protocol for IPv6	M	Managed Address Configuration
NDP	Neighbor Discovery Protocol	O	Other Configuration
mDNS	Multicast DNS	L	On-Link
LLMNR	Link-Local Multicast Name Resolution	RS	Router Solicitation
RA	Router Advertisement	NS	Neighbor Solicitation
MP6	Multicast Ping6	NA	Neighbor Advertisement
MLD	Multicast Listener Discovery	HTTP	Hypertext Transfer Protocol
SLAAC	Stateless Address Autoconfiguration	LAN	Local Area Network
NBNS	NetBIOS Name Service	WAN	Wide Area Network
Browser	Microsoft Windows Browser	LMA	Local Master Announcement
DNS-SD	Domain Name System Service Discovery	HA	Host Announcement
UPnP	Universal Plug and Play	DUID	DHCP Unique Identifier
5G	5th Generation Mobile Communication Technology	NetBIOS	Network Basic Input/Output System
IoT	Internet of Things	EUI-64	64-bit Extended Unique Identifier
DPDK	Data Plane Development Kit	BGP	Border Gateway Protocol
CPU	Central Processing Unit	GHz	GigaHertz
GB	GigaByte	DAD	Duplicate Address Detection
ARP	Address Resolution Protocol	IDS	Intrusion Detection System
ICMP	Internet Control Message Protocol	DoS	Denial-of-Service

## Analysis of on-link IPv6 address scanning technologies

IPv6 address scanning underpins key tasks such as network measurement^9^, topology discovery^10^, security analysis^11,12^, and host fingerprinting^13^. However, traditional exhaustive methods are impractical due to the vast and sparse IPv6 address space.

**Fig. 2:**
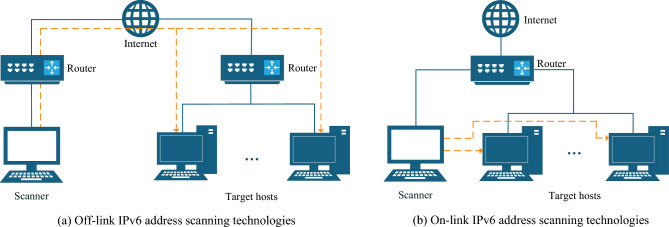
Categories of IPv6 address scanning technologies.

To improve efficiency, various IPv6 address scanning technologies have been proposed for different network settings, generally categorized as off-link and on-link based on the scanner’s location, as shown in Fig. 2. This paper focuses on on-link IPv6 address scanning technologies, which are essential for discovering on-link IPv6 hosts unreachable by off-link IPv6 address scanning technologies.

### Comparison of IP address scanning paradigms

Before analyzing specific IPv6 address scanning technologies, it is crucial to address a fundamental question: Can mature design principles from IPv4 address scanning be effectively translated to IPv6-only environments? The answer is largely negative due to the architectural divergence between the two protocols. In IPv4 address, scanning often relies on Broadcast mechanisms (e.g., ARP) and brute-force method (e.g., ICMP Echo Request). However, these principles are inapplicable in IPv6 for two reasons:

- Address space sparsity: The transition from 32-bit to 128-bit addresses makes brute-force scanning computationally infeasible.
- Protocol evolution: IPv6 deprecates Broadcast in favor of Multicast. Consequently, tools relying on broadcast-based discovery (such as those using ARP or NetBIOS in IPv4) fail completely in IPv6-only networks.

### Off-link IPv6 address scanning technologies

Off-link IPv6 address scanning addresses the challenge of the vast and sparse IPv6 address space through IPv6 target generation algorithms. Early technologies relied on DNS queries to collect limited IPv6 addresses. For example, Strowes et al.^14^ proposed a method that first performed reverse lookups using IPv4 addresses and then queried AAAA records to obtain IPv6 addresses. Subsequently, Fiebig et al.^15,16^ further utilized DNS reverse queries to gather IPv6 addresses. However, these IPv6 address collection methods were inefficient and yielded a limited number of IPv6 addresses.

To solve this, researchers have developed a series of IPv6 target generation technologies. Based on the generation approach, these technologies fall into two main categories: Those based on address structure information and those based on address statistical information. IPv6 target generation technologies based on address structure information (e.g., 6Gen, 6Tree, 6Hit, 6Graph, DET, AddrMiner, 6Probe, 6Hound)^17–25^ leverage entropy partitioning and hierarchical clustering to identify dense regions in the address space and guide scanning. IPv6 target generation technologies based on address statistical information (e.g., Entropy/IP, 6GCVAE, 6VecLM, 6Former, 6Vision, 6Diffusion)^13,26–29^ utilize deep learning and generative models to learn and predict address patterns from seed datasets, advancing scanning toward automation and intelligence.

Although these approaches greatly enhance efficiency and enable large-scale network measurement and security research, they exhibit limitations when applied to on-link network management. They rely on probabilistic models to predict GUA addresses and cannot discover LLA addresses, which are critical for on-link communication. Given the local scope and limited visibility of on-link IPv6 addresses, specialized technologies tailored to their unique characteristics are still necessary. The next section explores such on-link IPv6 address scanning technologies.

### On-link IPv6 address scanning technologies

Based on the methods of obtaining IPv6 addresses, existing on-link IPv6 address scanning technologies can be categorized into two categories: On-link IPv6 address scanning technologies based on IPv6-only information and on-link IPv6 address scanning technologies based on dual-stack correlation information. The former actively triggers host responses, while the latter obtains IPv6 addresses utilizing dual-stack correlation information, improving detection rates and system compatibility.

#### On-link IPv6 address scanning technologies based on IPv6-only information

This class of on-link IPv6 address scanning technologies typically leverage native IPv6 protocols. By sending probe messages, they elicit responses from on-link hosts containing their IPv6 address configurations to the scanning host, thereby completing the IPv6 address scanning.

There are currently four types of on-link IPv6 address scanning technologies, including MP6 scanning^1^, IEH scanning^2^, MLD scanning^3^, and SLAAC scanning^4^.

**Fig. 3:**
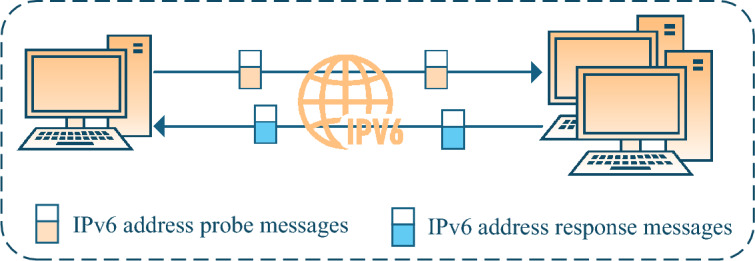
On-link IPv6 address scanning technologies based on IPv6-only information.

As shown in Fig. 3, these technologies involve sending various types of IPv6 probe messages to ff02::1 (link-local scope all-nodes multicast address), such as ICMPv6 Echo Request messages, ICMPv6 Echo Request messages with IEH headers, MLD Query messages, RA messages, to induce alive on-link hosts to send response messages or error messages, from which the host’s IPv6 address can be extracted.

However, as IPv6 address security awareness increases, major OS vendors and on-link administrators may configure corresponding security policies to intercept or discard probe messages, and the aforementioned IPv6 address scanning technologies also face the risk of being blocked by firewalls or layer-2 switches.

#### On-link IPv6 address scanning technologies based on dual-stack correlation information

**Fig. 4:**
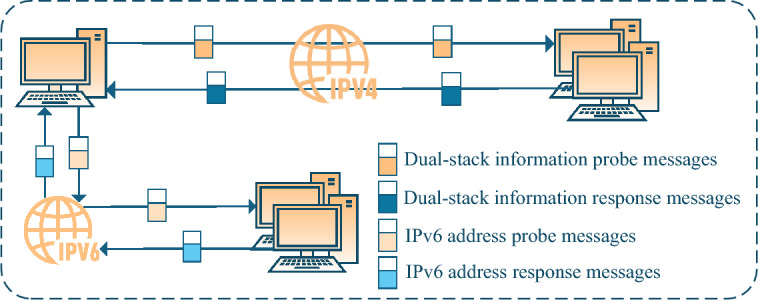
On-link IPv6 address scanning technologies based on dual-stack correlation information.

As shown in Fig. 4, on-link IPv6 address scanning technologies based on dual-stack correlation information leverage the widespread deployment of IPv4/IPv6 dual-stack hosts. By extracting shared identifiers—such as hostnames, MAC addresses, and DNS names—via IPv4 protocols, and mapping them to IPv6 addresses using relevant protocols, this approach circumvents the limitations of IPv6-only scanning, which often suffers from probe messages interception.

Representative technologies include LinkScan6^6^ and LLMNR6^7^, which obtain hostnames via NBNS protocol and discover IPv6 addresses through mDNS or LLMNR protocol queries. FScan6^8^ extends compatibility across operating systems by leveraging the Browser and DNS-SD protocols. Other technologies use UPnP protocol^30^ and mDNS protocol^31^ to induce responses that reveal IPv6 addresses, relying on service discovery mechanisms or device self-identification.

Despite their effectiveness in certain contexts, these technologies face limitations: On-link IPv6 address scanning technologies based on IPv6-only information are vulnerable to packet filtering, while on-link IPv6 address scanning technologies based on dual-stack correlation information depend on specific protocol support. As IPv6 adoption and security policies evolve, existing technologies must adapt. The following section analyzes current limitations and practical challenges in on-link IPv6 address scanning technologies.

### Limitation analysis of existing on-link IPv6 address scanning technologies based on IPv6-only information

Existing on-link IPv6 address scanning technologies based on IPv6-only information face the following issues due to limitations in IPv6 address protection policies and the detection methods employed.

#### Low completeness of IPv6 address scanning

Due to the vast IPv6 address space, network nodes typically possess multiple IPv6 addresses, including one LLA address, several permanent GUA addresses, and associated temporary GUA addresses. However, existing on-link IPv6 address scanning technologies based on IPv6-only information are limited in scope. MP6 scanning identifies only one IPv6 address per node–LLA addresses on CentOS and GUA addresses on Ubuntu. IEH scanning uses invalid extension headers to detect GUA addresses on Ubuntu and LLA addresses on CentOS 8. MLD scanning achieves better address coverage, responding to various Windows Server and Ubuntu versions, but retrieves only LLA addresses. SLAAC scanning is similarly restricted to scanning LLA addresses across all tested OSs.

#### Insufficient OS coverage

Due to variations in IPv6 protocol stack implementations across major OS vendors, on-link IPv6 address scanning technologies based on IPv6-only information often suffer from limited OS coverage, as different systems handle probe messages inconsistently. For instance, MP6 scanning, IEH scanning, MLD scanning, and SLAAC sacnning each scan fewer than 12 OS types in the setup described in “Comparative analysis of experimental results”. MP6 scanning and IEH scanning are ineffective on Windows nodes, which block external ICMPv6 messages by default. Although MLD scanning uses MLD Query messages and most OSs enable MLD snooping by default, some OSs (e.g., Windows 10/11, Windows Server 2012, CentOS 8) require explicit triggering. SLAAC scanning covers all OS types but effectively detects only up to 8 OSs.

### Limitation analysis of existing on-link IPv6 address scanning technologies based on dual-stack correlation information

Existing on-link IPv6 address scanning technologies based on dual-stack correlation information can bypass firewall and layer-2 switches filtering, enabling discovery of LLA, permanent GUA, and temporary GUA addresses. However, they suffer from limited OS compatibility. For example, UPnP-based methods fail on modern OSs lacking UPnP support, while mDNS and LLMNR6 offer partial coverage–mainly Windows platforms–with limited or no macOS/Linux support. AScan6 targets macOS but lacks broad OS applicability.

FScan6 improves coverage by combining active scanning and passive monitoring, supporting 26 OS versions across Windows, Linux, and macOS while capturing all IPv6 address types^8^. Nonetheless, dual-stack reliance remains a critical limitation, especially in IPv6-only environments, where such methods are inefficient or inapplicable.

#### Low IPv6 address scanning efficiency

IPv6 address scanning efficiency is typically measured by the number of alive addresses discovered per unit time, reflecting a tool’s discovery performance. On-link IPv6 address scanning technologies based on dual-stack correlation information (e.g., LinkScan6, LLMNR6) offer fast scanning, average completion time < 8 seconds (s), but suffer from limited OS support, significantly reducing the number of discoverable addresses and thus overall efficiency.

FScan6, the most advanced on-link IPv6 address scanning technologies, achieves broader OS coverage. However, its use of the Browser protocol introduces protocol-level delays: Responses are randomly distributed over a 30-second window, necessitating a minimum wait time of 30s to collect all replies. This design greatly prolongs each scan, ultimately reducing scanning efficiency.

#### Cannot operate in IPv6-only networks

As IPv6-only network become a core component of Next-Generation Internet infrastructure, their deployment is accelerating globally. In China, the Central Cyberspace Administration, together with other key agencies, released the “Key Points for Deepening the Large-Scale Deployment and Application of IPv6 by 2025”^32^, emphasizing the transition to IPv6-only in critical domains such as information infrastructure, 5G, and IoT.

Traditional on-link IPv6 address scanning technologies based on dual-stack correlation information are increasingly misaligned with this trend. These technologies depend on IPv4/IPv6 protocol interactions and are inherently unsuitable for IPv6-only environments, limiting their efficiency and adaptability.

To overcome these limitations, IPv6 address scanning technologies must move beyond dual-stack dependency and explore native IPv6 mechanisms. Among them, hostname-based IPv6 address scanning offers strong potential due to its protocol independence and compatibility with IPv6-only networks. By leveraging hostnames as stable network identifiers, this approach provides a robust foundation for on-link IPv6 address scanning. The following sections detail its underlying principles and technical advantages.

To clarify the the novelty and distinction of the proposed HFinder6 compared to existing technologies, Table 2 compares the discovery mechanisms, protocol dependencies, and limitations with those of existing technologies.

**Table 2:** Side-by-side comparison of mechanisms between HFinder6 and mainstream on-link IPv6 address scanning technologies.

Categories	Technologies	Core protocols used	Discoveries mechanisms
IPv6-only information	HFinder6	NDP + DHCPv6	Active triggering (RA M=1)
	MP6	ICMPv6	Multicast echo request
	IEH	ICMPv6	Probe with invalid extension header
	MLD	MLD	Multicast listener query
	SLAAC	NDP	MAC address + prefix prediction
Dual-stack correlation information	LinkScan6	NBNS	IPv4 broadcast $\rightarrow$ mDNS/LLMNR
	LLMNR6	NBNS	IPv4 broadcast $\rightarrow$ LLMNR
	FScan6	Browser	IPv4 broadcast $\rightarrow$ mDNS/LLMNR

Notably, while existing technologies such as LinkScan6 and FScan6 also utilize hostname information, they rely on IPv4-specific protocols for hostname discovery. In contrast, HFinder6 operates strictly within the IPv6 protocol stack. This ensures full functionality in IPv6-only environments, where on-link IPv6 address scanning technologies based on dual-stack correlation information fail.

## Basics of IPv6 address scanning mechanism based on hostname correlation

This section systematically explains the theoretical foundations and technical principles of the IPv6 address scanning mechanism based on hostname correlation. As a key technical approach for on-link IPv6 address scanning, the hostname correlation method extracts hostname identifiers from network protocols to establish a mapping between hostname representations and IPv6 addresses, thereby providing target clues for subsequent IPv6 address scanning.

### Fully qualified domain name

According to RFC 4704^33^ guidelines, when a host uses the DHCPv6 protocol to send a Solicit message to a DHCPv6 server, it typically includes its FQDN using DHCPv6 Option 81, enabling the DNS to maintain the mapping between the host’s FQDN and the assigned IPv6 address.

**Fig. 5:**
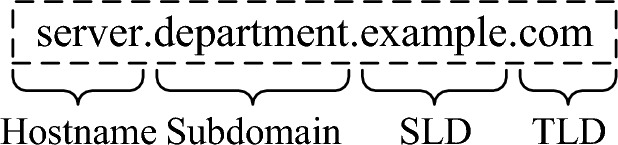
FQDN structure diagram.

The domain name field in the FQDN option contains the entire or partial FQDN of the DHCP client, which include the hostname, subdomain, SLD, TLD, and root domain, where the root domain is omitted by default, as shown in Fig. 5. If the client only aware of a portion of its FQDN, the request it sends contains an unqualified name, i.e., the hostname, because the client only knows part of the name when sending the request but may not know the zone in which the name will be embedded. Within on-link network, hostnames are typically unique to identify different hosts and services.

### Stateful address autoconfiguration mechanism

IPv6 address configuration is primarily categorized into SLAAC and stateful address autoconfiguration (DHCPv6). Unlike SLAAC, where hosts autonomously generate addresses without central management, the DHCPv6 protocol retains the automated management capabilities of IPv4 DHCP while introducing enhancements tailored to IPv6, effectively addressing the inherent limitations of SLAAC. While SLAAC offers efficiency, it lacks inherent security mechanisms. To address vulnerabilities such as DAD flooding, recent research has introduced optimized SLAAC addressing strategies. These methods incorporate hidden or secure DAD mechanisms to mitigate DoS threats in specific environments like drone networks^34–36^.

As an integral component of the IPv6 protocol stack, DHCPv6 enjoys broad applicability across modern OSs. Its implementation is typically embedded within the protocol stack by vendors, eliminating the need for user configuration and effectively resolving issues such as low OS coverage and incompatibility with IPv6-only networks. According to RFC 8415^37^, DHCPv6 operates over UDP ports 546 (client) and 547 (server), allowing hosts to dynamically obtain addresses and configuration parameters from a server. This mechanism works in conjunction with the NDP protocol, where flag bits in RA messages determine the host’s configuration behavior. Specifically, when the M flag in an RA message is set to 1, the host is mandated to obtain a stateful IPv6 address via the DHCPv6 protocol.

### NDP protocol

NDP protocol, as the core protocol of IPv6, performs critical tasks such as address resolution, router discovery, and neighbor state maintenance in IPv6 networks. NDP protocol includes five message types: RS messages, RA messages, NS messages, NA messages, and Redirect messages.

According to the guidelines in RFC 4861^38^ and RFC 3315^39^, when the M flag bit in the RA message is set to 1, the host must utilize DHCPv6 to obtain an IPv6 address. That is, regardless of whether a DHCPv6 server exists within the link, the client will send a Solicit message to the DHCPv6 server to request address allocation.

## Efficient IPv6 address scanning technology based on hostname correlation in IPv6-only network

Through the problem definition and based on an analysis of hostname correlation, this section proposes an efficient IPv6 address scanning technology based on hostname correlation in IPv6-only network.

### Problem definition and threat model

#### Problem definition

Let $\mathbb {N}$ denote on-link active hosts *H* in IPv6-only network. Each host *h* has IPv6 addresses *A* and a hostname *N*. The objective is to discover the mapping $S = \{ (A, N) \mid h \in H \}$. We aim to maximize the discovered set |*S*| and minimize scanning time *T* within a sparse address space.

#### Threat model

The threat model assumes the scanner is an authorized on-link node facing the following constraints: First, the network lacks IPv4 traffic, precluding broadcast discovery. Second, targets (e.g., Windows) drop unsolicited ICMPv6 probes by default. Finally, targets generally adhere to NDP and DHCPv6 standards and respond to multicast request.

### Feature analysis of hostname correlation

To justify the use of hostname correlation information in implementing IPv6 address scanning within on-link network, this section analyzes the uniqueness of hostname across mainstream OSs and examines how hostname correlation enables the mapping between hostnames and IP addresses.

(1) Within an on-link network, hostnames are unique in modern OSs. As a core component of the Internet infrastructure, DNS services are deeply integrated into all modern OSs. Hostnames, as fundamental elements of DNS, serve to identify specific devices or services within the link. Within the same local network, to prevent naming conflicts, the uniqueness of hostnames across different nodes is enforced by either: Manual configuration during OS installation, system-level conflict detection (e.g., Windows NetBIOS conflict detection, Linux Avahi hostname probing), or DHCPv6/DNS-SD dynamic update mechanisms when available.

(2) Hostnames are universally used in network protocols. As one of the fundamental identifiers of network devices, hostnames occupy a foundational role in modern network protocol architectures. From a protocol stack perspective, hostnames span multiple layers of the network protocol stack: At the data link layer, the NDP protocol uses hostname for address resolution; At the network layer, the DHCPv6 protocol utilizes hostname for address allocation management; At the application layer, protocols such as DNS and HTTP rely on hostname for service addressing. Because these protocols are implemented by OS vendors rather than user applications, hostname embedding behavior occurs automatically and consistently, which is a key prerequisite for the IPv6 address scanning technology to operate without requiring any configuration on target hosts.

(3) Hostname correlation can be used to establish a mapping relationship between hostnames and IP addresses. As specified in RFC 4704^33^, IPv6 clients can use FQDN to notify the server to maintain the mapping between hostnames and addresses. When present, the structure of this field allows extraction of the hostname portion even if a complete FQDN is not provided. While obtaining the hostname information of a host, this mapping can likewise be used to retrieve the host’s IPv6 address configuration. This essentially achieves on-link IPv6 address scanning through the dynamic binding of domain names and IP addresses, facilitated by protocol coordination mechanisms.

### Summary and problem analysis of existing hostname acquisition methods

Existing on-link IPv6 address scanning technologies based on dual-stack correlation information typically use protocols such as DNS-SD and Browser to obtain hostname. DNS-SD protocol is a service discovery mechanism built upon the standard DNS protocol, enabling devices to discover available network services within LANs or WANs through DNS queries. The DNS-SD protocol constructs a DNS-SD Query message named “_services._dns-sd._udp.local”, then receive a response containing the service names of alive on-link hosts, and parse the hostname from it^40^. However, constructing DNS-SD Query messages relies on the host’s IPv4 address and MAC address, so it is not compatible with the IPv6-only address scanning technology proposed in this paper.

The Browser protocol as a core mechanism for service discovery and resource enumeration in Windows-based networks, primarily used for identifying hosts and services within LANs. Hosts within the network periodically send LMA messages to request other hosts to send HA messages, thereby maintaining an updated resource list. Newly joined hosts also send HA messages to announce they presence. Hostnames can be derived from the NetBIOS names contained in these HA messages. However, constructing HA messages requires sending request messages with the destination IPv4 address set to the IPv4 broadcast address (255.255.255.255), so this method does not work in IPv6-only networks. Additionally, HA messages are randomly sent within a 30-s time window, which reduces the efficiency of IPv6 address scanning.

In summary, existing hostname acquisition methods suffer from several critical limitations, including dependency on the IPv4 protocol stack (e.g., DNS-SD, Browser, and NBNS), unpredictable timing mechanisms (such as randomized broadcasts in the Browser protocol), insufficient OS coverage (e.g., lack of NBNS support in Linux and strict firewall restrictions in Windows), and the absence of active triggering mechanisms in IPv6-only networks. These shortcomings motivate the need for an IPv6-only, actively triggered, and cross-OS-compatible hostname acquisition methods.

### Feasibility analysis of using DHCPv6 protocol to obtain hostnames of alive nodes

The analysis presented in “Hostname correlation fature analysis” demonstrates, that hostnames exhibit unique and universal properties, alongside the capability to establish mappings with IP addresses, thereby serving as ideal identifiers for on-link IPv6 address scanning. However, as highlighted in “Summary and problem analysis of existing hostname acquisition methods”, the current methods for obtaining hostnames in on-link IPv6 address scanning technologies have significant shortcomings. Consequently, there is an urgent need to develop an efficient hostname acquisition method that can operate in IPv6-only network.

Within this context, the NDP protocol, as a foundational component of the IPv6 network architecture, presents a potentially viable approach. As defined in the relevant RFCs, the RA messages in the NDP protocol contain three critical flags: The M flag, the O flag, and the L flag. These flags collectively control the address configuration strategy and network behavior of IPv6 nodes. Experimental validation confirms that even when the DHCPv6 server is absent (a common scenario in IPv6-only networks) hosts still send Solicit messages to UDP port 547 as mandated by RFC 8415^37^ whenever the M flag in RA messages is set to 1. This guarantees that hostname information can be obtained regardless of DHCPv6 server deployment.

**Fig. 6:**
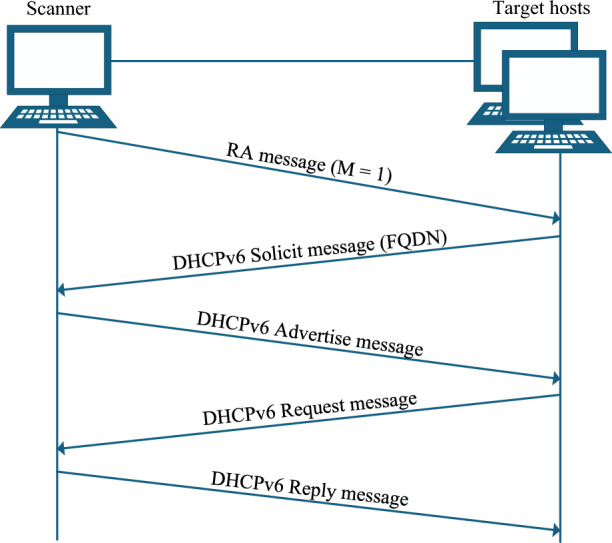
The workflow of the NDP protocol when the managed address configuration flag is set (M = 1).

As shown in Fig. 6, based on an understanding of the aforementioned protocol mechanisms and further validated through empirical testing, scanner actively sending RA messages with the M flag set to 1 in the target network can induce scanned hosts to initiate the DHCPv6 address request process. During this process, the target hosts typically sends back a Solicit message containing the FQDN field to the scanner. By parsing this FQDN field, the hostname information of the target node can be extracted. To ensure complete capture of Solicit messages, the scanner must wait long enough after sending RA messages. As shown in Table 3, experiments demonstrate that 80% of hosts send Solicit within $< 150$ ms and the slowest observed delay among all OS versions is 340 ms. Furthermore, according to RFC 8415^37^, the minimum retransmission time for Solicit messages is 1 second. Therefore, selecting a 1 second capture window ensures the acquisition of the initial response while avoiding the redundancy of retransmissions.

**Table 3:** DHCPv6 solicit response delays across different OSs (average of 3 runs).

OSs	Solicit delays (ms)	OSs	Solicit delays	OSs	Solicit delays
Windows 7	114	Windows Server 2019	134	Ubuntu 20.04	20
Windows 10	97	Windows Server 2022	340	Ubuntu 22.04	48
Windows 11	121	Ubuntu Server 20.04	32	CentOS 8	19
Windows Server 2012	156	Ubuntu Server 22.04	17	CentOS 9	43

Compared to existing methods based on dual-stack correlation information for obtaining hostname, this method offers the significant advantage of operating independently of IPv4 infrastructure, as it relies solely on the IPv6 protocol suite to extract hostname information. Moreover, owing to the broad support for stateful address autoconfiguration mechanisms in current mainstream OSs, this method demonstrates excellent adaptability and universality across different system environments. In summary, this hostname acquisition mechanism, which induces DHCPv6 Solicit messages based on RA flag bits and extracts the FQDN field, effectively addresses the shortcomings of existing technologies in IPv6-only environment, thereby providing a novel technical approach and support method for on-link IPv6 address scanning.

**Fig. 7:**
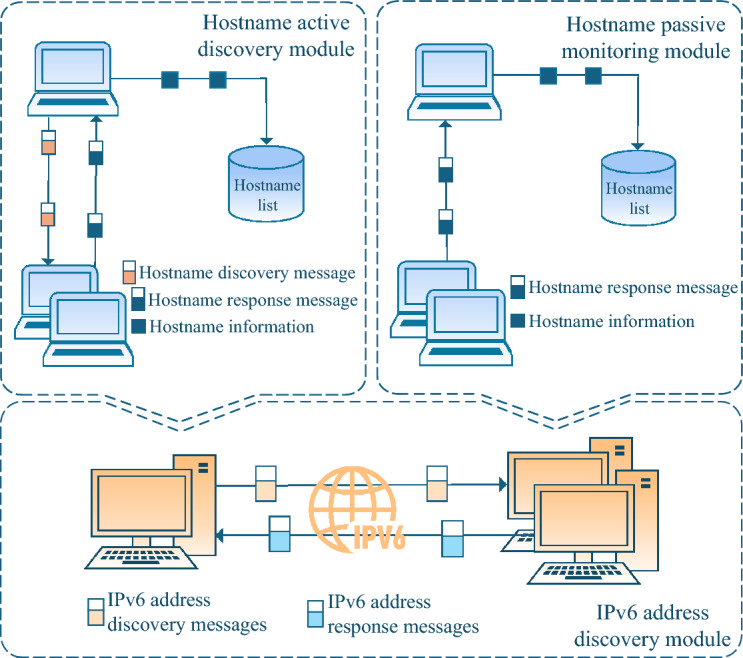
Main process of efficient IPv6 address scanning technology based on hostname correlation in IPv6-only network.

Based on the above analysis, this section addresses the issues of low completeness in single-node IPv6 address scanning, insufficient OS coverage in existing on-link IPv6 address scanning technologies based on IPv6-only information, and the problems of lengthy scanning processes and inability to operate in IPv6-only network in on-link IPv6 address scanning technologies based on dual-stack correlation information. This section explores the use of protocol collaboration to actively trigger the DHCPv6 protocol and combine it with the mDNS protocol and LLMNR protocol, proposing an efficient on-link IPv6 address scanning technology based on hostname correlation that combines active discovery and passive listening. This technology is divided into 3 modules: Hostname active discovery module, hostname passive monitoring module, and IPv6 address discovery module. The overall process is shown in Fig. 7.

### Uniqueness of the mechanism and differentiation from RA spoofing

Notably, the theoretical mechanism described above, using RA messages to trigger DHCPv6 responses, shares similarities in underlying packet interaction with RA spoofing techniques for Man-in-the-Middle threats. However, the approach proposed in this paper differs fundamentally in its design principles. While RA spoofing tools aim to hijack traffic^41^, this study repurposes the mechanism for benign network measurement. The specific distinction lies in the single-probe strategy and stateless listening logic (i.e., not completing the DHCPv6 handshake) introduced in our subsequent HFinder6 implementation. These measures transform a potential threat into a controlled, safe, and efficient scanning technique. The theoretical mechanism for the purpose of hostname discovery has not been explored in prior IPv6 address scanning literature.

### Hostname active discovery module

**Fig. 8:**
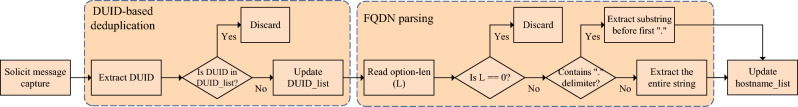
DUID-based deduplication and FQDN parsing workflow diagram.

This module leverages the NDP and DHCPv6 protocols to actively discover hostname. Building on the principle described above, a network interface is first selected, the RA message construction were implemented using the Scapy tool (as detailed in the ‘Experimental Setup’). The RA message is encapsulated in an IPv6 header with the destination address set to the link-local all-nodes multicast address (ff02::1). Crucially, the M flag in the RA message is set to 1 to mandate stateful configuration. Additionally, prefix information is appended with the 2001:db8::/64 prefix, where O and L flags are set to 1 to simulate a standard network environment. According to RFC 3849^42^ guidelines, the network prefix 2001:db8::/64 serves as an exemplary configuration. The protocol interaction follows a strictly defined timing sequence. Upon multicasting the RA message, the scanner immediately initiates a listening window on UDP port 547 (the default DHCPv6 server port). As shown in Table 3, most OSs respond within milliseconds, a 1-second capture window is enforced. During this window, the scanner captures Solicit messages. To avoid duplicate captures, the module deduplicates Solicit messages using the extracted DUID. The hostname of the target host can be parsed from the FQDN field of the DHCPv6 Solicit message, as shown in Algorithm 1. Figure 8 illustrates the detailed process of DUID-based deduplication and FQDN parsing. It is important to note that the scanner does not complete the DHCPv6 handshake (i.e., no Advertise or Reply messages are sent back to the targets). This “fire-and-listen” approach ensures the scanner extracts the FQDN and DUID information without establishing actual stateful bindings or interfering with the target’s IP configuration.

**Algorithm 1:**
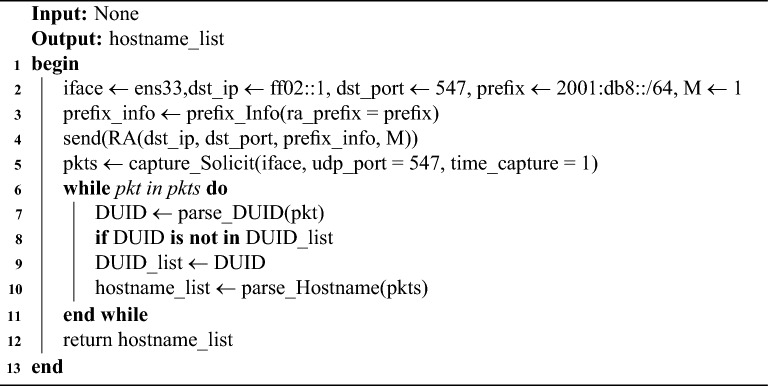
Active discovery algorithm for on-link hostnames.

### Hostname passive monitoring module

Existing on-link IPv6 address scanning technologies typically rely on scan results at a specific point in time to identify IPv6 addresses within on-link network. However, these technologies are incapable of promptly detecting the subsequent addition of hosts or the renewal of IPv6 addresses by alive hosts, thus falling short of comprehensively fulfilling the objective of on-link IPv6 address discovery.

This paper guided by the specifications outlined in RFC 8415^37^ and utilizes the characteristics of the DHCPv6 protocol. Specifically, when a node with DHCPv6 enabled joins the network, it is required to send a Solicit message to the DHCPv6 server within on-link to initiate address allocation. Although on-link nodes typically use the SLAAC IPv6 allocation method and does not deploy a DHCPv6 server, this does not affect the node’s behavior of sending Solicit messages.

Based on the above principles, the hostname passive monitoring module does not send probe messages but continuously monitors UDP port 547. When subsequent nodes or hosts joining the link send Solicit messages for IPv6 address lease renewal, this module captures these messages, performs deduplication based on DUID, then passes them to the on-link address active discovery module for message analysis. It extracts the hostname information contained in the FQDN field and adds it to the hostname list, as shown in Algorithm 2.

**Algorithm 2:**
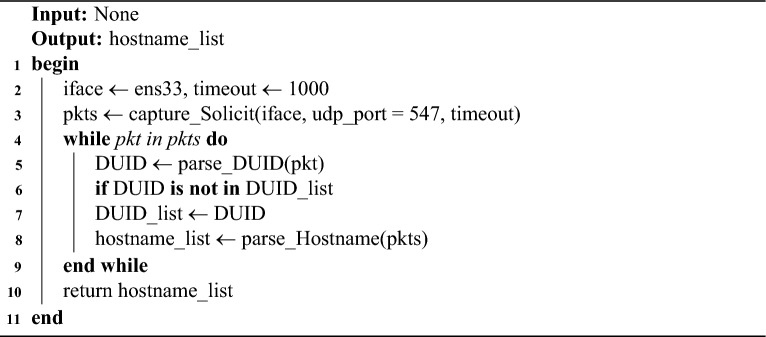
Passive monitoring algorithm for on-link hostnames.

### IPv6 address discovery module

This module utilizes the hostname information of hosts within the link to construct probe messages and subsequently obtains the corresponding IPv6 address configuration by parsing the response messages. Currently, the main protocols that can perform the above tasks are the mDNS protocol and the LLMNR protocol.

mDNS is a zero-configuration protocol operating over UDP port 5353, enabling hostname and service IP resolution within an on-link network without relying on traditional DNS servers. Protocol analysis and testing show that mDNS is natively supported in most Linux distributions (e.g., Ubuntu, CentOS), but unsupported in earlier Windows versions (e.g., Windows 7, Windows 8).

LLMNR protocol, proposed by Microsoft, allows hostname resolution via IPv4/IPv6 multicast in local networks without DNS. Empirical evaluation shows LLMNR is well-supported on Windows, while Linux compatibility is limited to specific distributions (e.g., Ubuntu 18.04).

To maximize OS compatibility and host discovery completeness, this study adopts a hybrid strategy that leverages both mDNS and LLMNR for concurrent address probing.

This module utilizes the hostname list provided by the two aforementioned hostname retrieval modules, appends the “.local” suffix to the hostnames in the list, and then constructs mDNS Query messages with the destination address set to the special multicast address ff02::fb, the destination port number set to 5353, and the query content set to $<\hbox {Hostname.local}>$. Simultaneously, it constructs LLMNR Query messages with a destination multicast address of ff02::1:3, a destination port number of 5355. When on-link hosts receive the probe messages, they send their network address configurations to the probe host, then the probing host categorizes the received IPv6 addresses into GUA and LLA based on their prefixes, and adds them to the IPv6 address list, as detailed in Algorithm 3.

**Algorithm 3:**
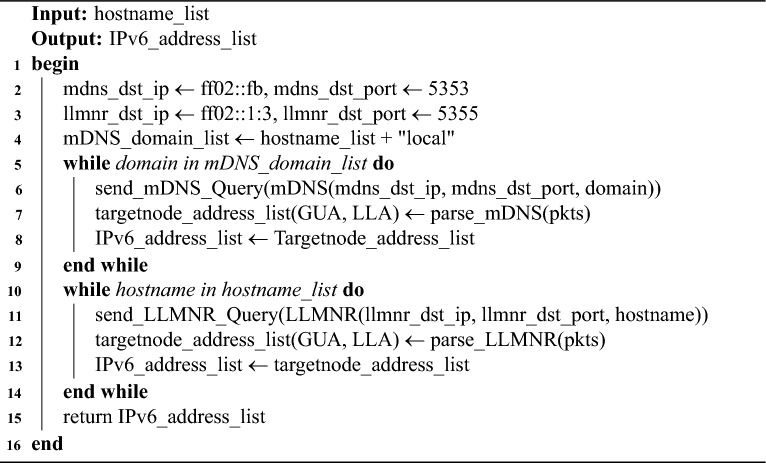
IPv6 address discovery algorithm.

## Comparative analysis of experimental results

This section first presents the details of the test environment setup, and then organize the experimental results of this paper around the four key research questions that this paper attempts to answer. These research questions are as follows:

OS coverage (RQ1): to what extent does HFinder6 support for mainstream OSs? Specifically, does HFinder6’s host discovery and IPv6 address scanning capabilities exhibit universality across different OS environments? IPv6 address scanning completeness (RQ2): when scanning on-link nodes, can HFinder6 obtain their complete IPv6 address configurations (including LLA address, permanent GUA address, and temporary GUA address)? How complete are the IPv6 address scanning results? IPv6-only network adaptability (RQ3): can HFinder6 function effectively in an IPv6-only network? Specifically, in the absence of IPv4 support, does its scanning performance consistent with that in a dual-stack environment? IPv6 address scanning efficiency (RQ4): compared to existing mainstream on-link IPv6 address scanning tools, does HFinder6 demonstrate a significant advantage in the number of alive IPv6 addresses it can discover within a given time frame?

### Experimental environment and test methods

Experimental setup: The scanning host is configured with an Intel Xeon Gold 6230 CPU, operating at a clock speed of 2.10 GHz, and 64 GB of memory. The scanning host runs Ubuntu 20.04. The scanning tool, HFinder6, was implemented using Python 3.9 and the Scapy 2.4.5 packet manipulation library. To establish a reliable ground truth, this paper executed local network configuration commands on each of the 20 target nodes individually to export their ground-truth IPv6 address lists.

**Fig. 9:**
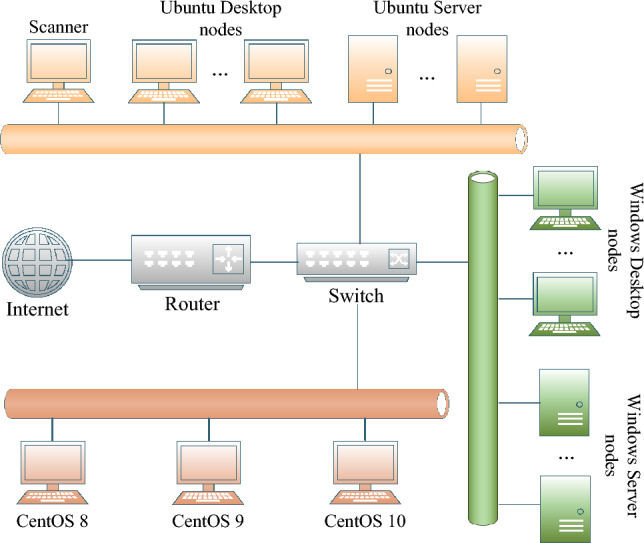
Network topology of experimental environment.

Environment setup: This paper sets up a total of 20 different OS versions, including 4 Windows Desktop versions (Windows 7, Windows 8, Windows 10, and Windows 11), 5 Windows Server versions (Windows Server 2012, Windows Server 2016, Windows Server 2019, Windows Server 2022, and Windows Server 2025), 3 CentOS versions (CentOS 8, CentOS 9, and CentOS 10), 3 Ubuntu Desktop versions (Ubuntu 20.04, Ubuntu 22.04, and Ubuntu 24.04), and 5 Ubuntu Server versions (Ubuntu Server 20.04, Ubuntu Server 21.04, Ubuntu Server 22.04, Ubuntu Server 23.04, and Ubuntu Server 24.04), to create an IPv6 typical on-link environment and conduct comparative experiments, as shown in Fig. 9.

Testing methodology: For RQ1, this paper established the local IPv6 network environment shown in Fig. 9, consisting of 20 different mainstream OSs, and ran the HFinder6 tool in this environment to evaluate the tool’s IPv6 address discovery capabilities across different OSs. For RQ2, this paper uses the ratio of the number of IPv6 addresses scanned by HFinder6 to the total number of IPv6 addresses as an indicator to assess the tool’s IPv6 address scanning completeness. For RQ3, the nodes in the established environment are not connected to the IPv4 network, and the tool proposed in this paper is run in an IPv6-only network environment to observe whether the results are unaffected. For RQ4, to minimise the impact of network fluctuations on IPv6 address scanning, this paper conducted five repeated experiments using HFinder6 in the aforementioned environment, taking the average scan time and comparing it with other on-link IPv6 address scanning scripts and tools.

### Experimental results and analysis

This paper evaluates the scanning capabilities of HFinder6 from four aspects: OS coverage (for RQ1), IPv6 address scanning completeness (for RQ2), IPv6-only network adaptability (for RQ3), and IPv6 address scanning efficiency (for RQ4). It is compared with mainstream on-link IPv6 address scanning scripts and tools, including four on-link IPv6 address scanning scripts based on IPv6-only information in Nmap tool (MP6 script, IEH script, MLD script, and SLAAC script) and three on-link IPv6 address scanning tools based on dual-stack correlation information (LinkScan6, LLMNR6, and FScan6).

#### OS coverage

To evaluate the OS coverage capabilities of HFinder6, the scanning host executes HFinder6 along with 7 mainstream IPv6 address scanning scripts and tools within the experimental environment illustrated in Fig. 9, and records the IPv6 address scanning results for different OS nodes for each script or tool.

**Fig. 10:**
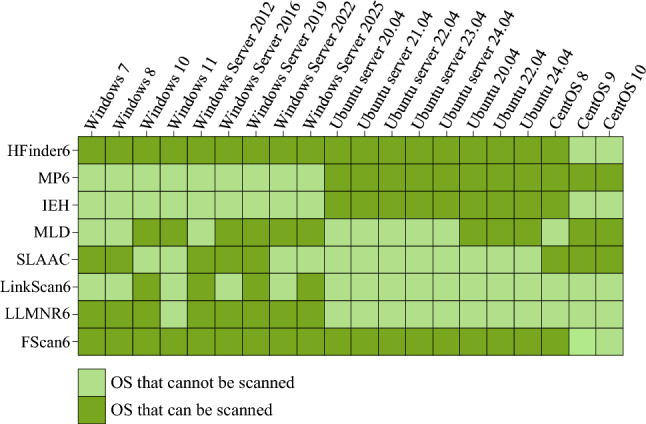
Experimental results showing the degrees of OS coverage for different scripts and tools.

As illustrated by the experimental results in Fig. 10, the IPv6 address scanning scripts based on IPv6-only information MP6 and IEH can effectively scan Linux nodes but exhibit limited support for Windows nodes. This limitation arises because both scripts use multicast ICMPv6 Echo Request messages to trigger responses from the scanned hosts, and Windows nodes’ firewalls default to blocking ICMPv6 Echo Request messages, while Linux processes ICMPv6 Echo Request messages in compliance with RFC 4291^43^. The SLAAC script theoretically covers all OS nodes, as most existing OSs support obtaining IPv6 addresses via SLAAC. However, the IPv6 addresses scanned by this script are constructed by combining the network prefix used within the link with the MAC address of the scanned host, resulting in estimated values that do not accurately represent the actual IPv6 addresses in most cases. According to experimental observations, the effective OS coverage achieved by SLAAC script is only 8.

Among on-link IPv6 address scanning tools based on dual-stack correlation information, LinkScan6 and LLMNR6 are capable of scanning IPv6 addresses only for Windows nodes, with limited or no support for Linux nodes. This is because both tools use the NBNS protocol to obtain the hostnames of alive hosts within on-link, and this method is not supported in Linux environments. In Windows nodes, LLMNR6 failed to scan Windows 11 because although the LLMNR protocol it uses is deployed on Windows 11, it is blocked by the firewall.

Compared to the currently most advanced on-link IPv6 address scanning tool, FScan6, HFinder6 demonstrates consistent OS coverage capabilities with FScan6 across different OSs and versions, covering 18 different OS versions, as shown in Fig. 10.

#### IPv6 address scanning completeness

To evaluate the IPv6 address scanning completeness of the technology proposed in this paper across different OSs and to compare it with other technologies, the scanning host executes HFinder6 alongside the aforementioned IPv6 address scanning tools in the experimental environment depicted in Fig. 9. The experiment detects all nodes with different OSs within the environment, computes the ratio of the number of IPv6 addresses scanned by each script or tool to the total number of IPv6 addresses in the environment, as defined by (1): Where $C_{addrscan}$ denotes the IPv6 address scanning completeness; $N_{addrscanned}$ represents the number of scanned addresses; $Sum_{addr}$ is the total number of addresses. The IPv6 address scanning completeness of each script or tool was then compared, as shown in Tables 4 and 5, where Table 4 presents the raw IPv6 address scanning results, and Table 5 summarizes the IPv6 address scanning completeness of each script or tool (the total number of IPv6 addresses in the experimental environment was 47).

1 $$\begin{aligned} C_{{addrscan}} = \frac{N_{{addrscanned}}}{{Sum}_{{addr}}} \times 100\% \end{aligned}$$

**Table 4:** Compatibility of on-link IPv6 address scanning scripts and tools across different OSs.

Operating systems	On-link IPv6 address scanning scripts and tools							
	HFinder6	MP6	IEH	MLD	SLAAC	LinkScan6	LLMNR6	FScan6
Windows 7	LLA/G1/G2$^{\textrm{a}}$	–	–	–	LLA	–	LLA/G1/G2	LLA/G1/G2
Windows 8	LLA/G1/G2	–	–	–	LLA	–	LLA/G1/G2	LLA/G1/G2
Windows 10	LLA/G1/G2	–	–	LLA	–	LLA/G1/G2	LLA/G1/G2	LLA/G1/G2
Windows 11	LLA/G1/G2	–	–	LLA	–	–	–	LLA/G1/G2
Windows Server 2012	LLA/G1	–	–	–	LLA	LLA/G1	LLA/G1	LLA/G1
Windows Server 2016	LLA/G1	–	–	LLA	LLA	–	LLA/G1	LLA/G1
Windows Server 2019	LLA/G1	–	–	LLA	LLA	LLA/G1	LLA/G1	LLA/G1
Windows Server 2022	LLA/G1	–	–	LLA	–	–	LLA/G1	LLA/G1
Windows Server 2025	LLA/G1	–	–	LLA	–	LLA/G1	LLA/G1	LLA/G1
Ubuntu 20.04	LLA/G1/G2	G1	G1	LLA	–	–	–	LLA/G1/G2
Ubuntu 22.04	LLA/G1/G2	G1	G1	LLA	–	–	–	LLA/G1/G2
Ubuntu 24.04	LLA/G1/G2	G1	G1	LLA	–	–	–	LLA/G1/G2
Ubuntu Server 20.04	LLA/G1	G1	G1	–	–	–	–	LLA/G1
Ubuntu Server 21.04	LLA/G1	G1	G1	–	–	–	–	LLA/G1
Ubuntu Server 22.04	LLA/G1	G1	G1	–	–	–	–	LLA/G1
Ubuntu Server 23.04	LLA/G1	G1	G1	–	–	–	–	LLA/G1
Ubuntu Server 24.04	LLA/G1	G1	G1	–	–	–	–	LLA/G1
CentOS 8	LLA/G1	LLA	LLA	–	LLA	–	–	LLA/G1
CentOS 9	–	LLA	–	LLA	LLA	–	–	–
CentOS 10	–	LLA	–	LLA	LLA	–	–	–

$^{\textrm{a}}$G1: permanent GUA address; G2: temporary GUA address.

As shown in Table 4, on-link IPv6 address scanning scripts based on IPv6-only information typically detect only the addresses of discoverable nodes. For example, MLD script uses multicast probe discovery in compliance with RFC 4291^43^, where multicast group members reply to ff02::1 broadcast with only the on-link addresses required for basic communication. In contrast, SLAAC script predicts addresses by combining the link’s network prefix with the MAC addresses of active nodes using the EUI-64 format, though these predicted addresses may not match the nodes’ actual on-link IPv6 addresses.

For scannable Windows nodes, on-link IPv6 address scanning tools based on dual-stack correlation information such as LinkScan6 and LLMNR6 can retrieve the full IPv6 address configuration by sending mDNS or LLMNR Query messages. As specified in RFC 6762^31^ and RFC 4795^44^, hosts with these protocols enabled return both LLA and GUA addresses in response. However, these tools depend on NBNS for dual-stack correlation, which is ineffective in Linux environments where the NetBIOS stack is disabled by default, preventing hostname queries and thus IPv6 address scanning for Linux nodes.

As presented in Table 5, HFinder6 exhibits superior IPv6 address scanning completeness, scanning at least 32 more addresses than on-link IPv6 address scanning scripts based on IPv6-only network. Its IPv6 address scanning completeness is 3.91 times that of MP6 script, 4.79 times that of IEH script, 3.91 times that of MLD script, and 5.37 times that of SLAAC script. Compared to dual-stack correlation infromation based on-link IPv6 address scanning tools, HFinder6’s IPv6 address scanning completeness is 4.79 times that of LinkScan6 and 2.26 times that of LLMNR6. Compared to the state-of-the-art FScan6, HFinder6 achieves the same IPv6 address scanning completeness as FScan6.

**Table 5:** Comparison of IPv6 address scanning completeness.

Scanning scripts and tools	Number of OSs	Number of IPv6 addresses	IPv6 address scanning completeness
HFinder6	18	43	91.4%
HFinder6	11	11	23.4%
IEH	9	9	19.1%
MLD	11	11	23.4%
SLAAC	8	8	17.0%
LinkScan6	4	9	19.1%
LLMNR6	8	19	40.4%
FScan6	18	43	91.4%

#### IPv6-only network adaptability

**Fig. 11:**
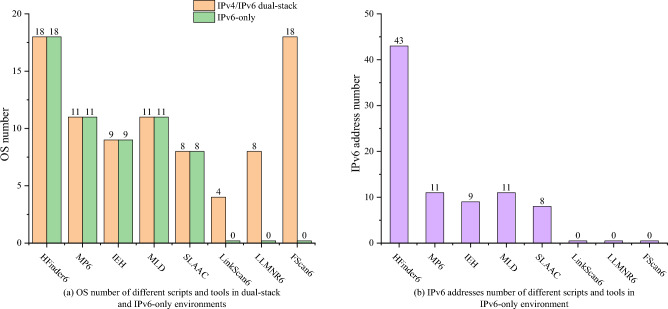
Experimental results showing the degrees of OS coverage and IPv6 address number for different scripts and tools.

To evaluate whether the technology proposed in this paper can operate normally within an IPv6-only network without relying on dual-stack network, this section isolates the 20 OS nodes depicted in Fig. 9 from the IPv4 network. The scanning host executes HFinder6 and other on-link IPv6 address scanning scripts and tools exclusively in IPv6-only network. The number of scanned OS nodes and IPv6 addresses is used as a reference, and the results are compared with those from an IPv4/IPv6 dual-stack network. The comparative outcomes are summarized in Fig. 11.

As illustrated in Fig. 11, the OS coverage and IPv6 address scanning results of HFinder6 within an IPv6-only network are identical to those in a normal dual-stack network. This is because HFind6 is based on the DHCPv6 and NDP protocol within the IPv6 protocol stack, while the mDNS protocol used for IPv6 address scanning employs both the IPv4 multicast address 224.0.0.251 and the IPv6 multicast address ff02::fb, which also support operation in an IPv6-only network. However, on-link IPv6 address tools such as LinkScan6, LLMNR6, and FScan6, which rely on dual-stack correlation information, become ineffective in IPv6-only networks. This is because they overly depend on IPv4 networks to obtain host information about alive hosts within the link.

#### IPv6 address scanning efficiency

**Fig. 12:**
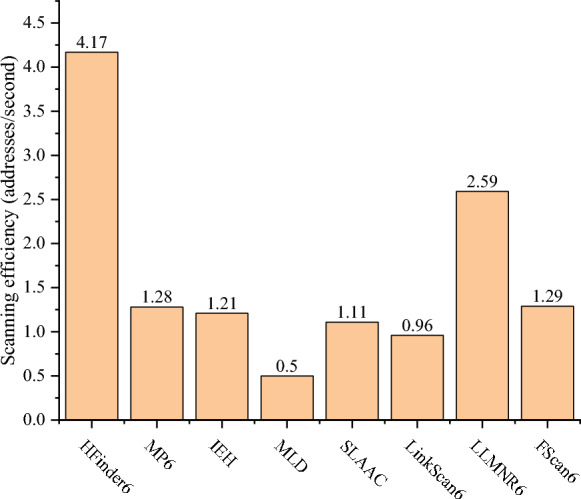
Comparison of IPv6 address scanning efficiency between different scanning scripts and tools.

To evaluate the IPv6 address scanning efficiency of HFinder6, this paper conducted five repeated experiments within the experimental environment depicted in Fig. 9, comparing HFinder6 with other on-link IPv6 address scanning scripts and tools to minimize the impact of network conditions on the experimental results. The efficiency metric was calculated as the ratio of the number of IPv6 addresses scanned to the time consumed by the scanning process, as defined by (2), where $E_{addrscan}$ denotes the IPv6 address scanning efficiency; $N_{addrscanned}$ represents the number of scanned addresses; $T_{addr}$ is the scanning duration. The IPv6 address scanning efficiency of each script and tool was compared, with the results shown in Table 6 and Fig. 12. Specifically, Table 6 lists the time consumed by each tool’s IPv6 address scanning process in the five repeated experiments, while Fig. 12 compares the IPv6 address scanning efficiency of each tool.

2 $$\begin{aligned} E_{{addrscan}} = \frac{N_{{addrscanned}}}{T_{{addr}}} \times 100\% \end{aligned}$$

**Table 6:** Time consumed by different scripts and tools in five experiments.

Scanning scripts and tools	Time consumed by IPv6 address scanning (s)					
	1st	2nd	3rd	4th	5th	Average time
HFinder6	10.28	10.24	10.36	10.27	10.31	10.29
MP6	8.58	8.62	8.59	8.62	8.54	8.59
IEH	7.48	7.39	7.44	7.41	7.45	7.43
MLD	22.14	22.45	20.58	21.73	22.09	21.79
SLAAC	7.21	7.06	7.26	7.16	7.26	7.19
LinkScan6	7.26	7.29	7.31	7.36	7.30	7.30
LLMNR6	7.39	7.36	7.39	7.31	7.28	7.35
FScan6	33.45	33.21	33.54	33.44	33.31	33.39

As shown in Table 6, on-link IPv6 address scanning scripts based on IPv6-only information generally achieve faster IPv6 address scanning speeds. The average IPv6 address scanning durations for the MP6, IEH, and SLAAC scripts are 8.59s, 7.43s, and 7.19s, respectively, whereas the MLD script requires a longer average of 21.79s. This difference arises because the protocol interactions of MP6, IEH, and SLAAC scripts follow a straightforward request-response model, enabling quicker scan completion. In contrast, the MLD script must wait for the MLD Query and Report interactions, resulting in a longer scanning time.

There exists significant differences in IPv6 address scanning speeds among on-link IPv6 address scanning tools based on dual-stack correlation information. The LinkScan6 tool and LLMNR6 tool can complete IPv6 address scanning in an average of 7.30s and 7.35s, respectively, while the HFinder6 tool requires an average of 10.29s to complete IPv6 address scanning. This is because the LinkScan6 tool and LLMNR6 tool use the NBNS protocol to directly query hostnames via IPv4 broadcasts without triggering additional protocol interactions, enabling shorter response times. The HFinder6 tool uses RA messages to induce hosts to send DHCPv6 Solicit messages containing hostnames, which inherently involves delays, as different OSs respond to RA messages at different rates, requiring more time to capture messages to avoid omissions. Additionally, the HFinder6 tool uses parallel queries via LLMNR and mDNS to obtain IPv6 addresses, which may cause target hosts to process multiple requests simultaneously, thereby delaying responses. The FScan6 tool, as the most advanced on-link IPv6 address scanning technology currently available, requires an average of 33.39s to complete IPv6 address scanning. This is because the FScan6 tool uses the Browser protocol to obtain local domain name information, and the target host responds to the Browser protocol randomly within a 30-second time window. As a result, the FScan6 tool must design a waiting period of at least 30s to capture all response messages.

As illustrated in Fig. 12, the IPv6 address scanning efficiency of the HFinder tool achieved the best performance among the 8 IPv6 address scanning scripts and tools tested, with an average rate of 4.17 addresses scanned per second. Although the state-of-the-art FScan6 tool excels in OS coverage and IPv6 address scanning completeness, its extended scanning duration leads to comparatively lower IPv6 address scanning efficiency. The LLMNR6 tool, despite having a IPv6 address scanning efficiency of 2.59, performs poorly in terms of OS coverage and IPv6 address scanning completeness.

Regarding network overhead, HFinder6 operates with minimal message interaction. The hostname discovery phase is triggered by a single multicast RA message, and the address discovery phase involves targeted unicast probes based on the discovered hostname list. Compared to exhaustive scanning, this deterministic interaction model occupies negligible network bandwidth.

Finally, regarding scanning efficiency in IPv6-only networks (RQ3), HFinder6 relies strictly on native IPv6 protocols. Consequently, its scanning performance remains consistent across both IPv6-only and dual-stack environments. To avoid redundancy, we omit a separate detailed analysis for the IPv6-only environment.

## Limitations and ethical considerations

This section outlines the technical limitations, operational safety measures, and ethical implications of the proposed approach.

### Technical limitations and deployment feasibility

HFinder6 relies on specific client behaviors that vary by environment. First, Some OSs (e.g., Windows XP, CentOS 7, MacOS) ignore the M flag in RA messages, resulting in scanning misses. Similarly, hosts with static IPs may drop multicast probes. Second, while administrators or protocols like NetBIOS usually enforce uniqueness of hostname, duplicate hostnames may occur due to obfuscation or misconfiguration. In such cases, the scanner might conflate distinct nodes into a single entity. This reduces the robustness of the mapping between hostnames and IP addresses in non-standard environments.

Conversely, in terms of deployment feasibility, HFinder6 demonstrates strong adaptability. Unlike tools relying on DNS servers, it parses hostnames directly from local packets, ensuring independence from infrastructure. Finally, its active multicast mechanism ensures compatibility with standard layer-2 switching environments.

### Operational safety strategies

Active RA injection carries potential risks. Unlike malicious tools, it limits its role to triggering Solicit messages and sends no replies. This ensures no IP addresses are assigned and no routing tables are modified. Additionally, the injected RA messages use low router preference and short lifetimes. This prevents overriding legitimate gateways, thereby ensuring the stability of production networks.

### Security and ethical compliance

From a security perspective, active RA traffic is distinct and detectable by IDS or RA Guard. Therefore, HFinder6 is designed strictly as an authorized audit tool, not for stealthy reconnaissance. Users must adhere to network security policies and obtain explicit authorization before deployment. Unauthorized use may trigger security alarms or violate compliance regulations regarding active probing.

## Conclusion

To address the limitations of existing on-link IPv6 address scanning technologies, namely, low completeness of IPv6 address scanning, insufficient OS coverage, low IPv6 address scanning efficiency and IPv4 dependency, this paper presents HFinder6. By actively triggering DHCPv6 Solicit messages and integrating NDP, mDNS, and LLMNR protocols, HFinder6 enables accurate and comprehensive host discovery in IPv6-only environments.

Experiments on 20 OS nodes show that HFinder6 achieves 91.4% address coverage across 18 mainstream OS versions, comparable to the state-of-the-art FScan6, while scanning over three times faster. These results validate its suitability for IPv6-only scenarios, such as enterprise intranets and IoT deployments.

Future work includes leveraging DPDK and kernel bypass to further enhance performance, employing machine learning for adaptive protocol selection, and constructing an IPv6 address knowledge graph using DNS and BGP data to broaden discovery capabilities.

## Data Availability

The datasets generated and analyzed during the current study are available from the corresponding author upon reasonable request.
